# Supplementary material for: Engaging Diverse Community Groups to Promote Population Health through Healthy City Approach: Analysis of Successful Cases in Western Pacific Region
Source: Int J Environ Res Public Health. 2021 Jun 19;18(12):6617. doi: 10.3390/ijerph18126617 (PMC8296388; doi:10.3390/ijerph18126617)
Supplement: Supplementary file 1 [file ijerph-18-06617-s001.zip › ijerph-1221741-supplementary.pdf]

Supplementary File S1. SPIRIT Model

**S***etting approach,* **S***ustainability*	The city should put a strong emphasis on developing activities in different settings such as schools, workplaces, and marketplaces to promote and influence health.<br>Has the City developed a strategic plan to ensure sustainable development of the city? Is the programme engaging in strategic partnerships with city management and planning processes? Are all the stakeholders in agreement on the main health and environment problems in the city? How would one secure the ownership of the programme to the community?
**P***olitical commitment,* **P***olicy,* **P***artnership*	The political leaders of the city should make a public commitment that they will move their cities towards becoming a Healthy City. There should be a written policy statement<br>Are health issues accorded high priority in public policy? Has the City involved all stakeholders, professionals, and academics with expertise in Healthy City to formulate healthy public policies? Is there a mechanism for wide consultation and regular review of the policies?<br>Is the city encouraging communities to participate in urban development for better health and quality of life?
**I***nformation*, **I***nnovation*	Information should be available in the following areas:<br>- important health problems and health issues in the city<br>- analysis of economic and social determinants of health for the city<br>- the concerns of health care delivery system of the city<br>- the special population groups at risk<br>- the existing health promotion programmes organised by different parties<br>- community perception of health<br>- level and standards of primary health care in the city<br>- The city should create a comprehensive health profile based on the above information, and then a city health plan can be developed.<br>- Programmes should be innovative in meeting the public needs and promote a climate to support changes. T<br>- Sharing ideas with others and publicise best practices.
**R***esources*, **R***esearch*	Is adequate resource earmarked for the programme? Has research framework been developed for needs assessment and measurement of outcomes? Has the city engaged experts in research?
**I***nfrastructure,* **I***ntersectoral*	There should be a steering committee with responsibility for the overall management and coordination of the programme. Does the steering committee include representatives of all the sectors and local stakeholders?<br>There should be a technical committee or working committee with members from different sectors to address specific projects. Does the committee have enough professional inputs?
**T***raining*	Are there training courses at different levels in health education and health promotion? The targets should be professionals, administrators, policy makers, politicians, and lay people. The city should identify institutions that can provide training for professionals, administrators, and the public. One must observe the spirit of capacity building in the community.

Supplementary File S2. Different Levels of Achievement of Healthy Cities in Western Pacific Region using SPIRIT Framework for AFHC Healthy City Awards

AFHC Award for a Healthy City with Good Infrastructure (Level 1)

(1) The city must demonstrate the initiatives in using multiple settings to promote population health with strategic planning.
(2) Political commitment to healthy public policy and involvement of different stakeholders are important steps forward.
(3) The city needs to create its city health profile with resources earmarked to address the health needs of the city, including training at different levels.
(4) The city should have the basic layout of good infrastructure and support and also involve different sectors.
(5) The city has laid down a good foundation of processes to help the city become a Healthy City.
(6) Many health-promotion activities have been launched to improve the health knowledge of the local citizens.
(7) Health services have been strengthened.

The next step should be engaging and empowering the community to build up a healthy environment and more health-promotion activities focussing on promoting wellness.

AFHC Award for a Healthy City with Good Dynamics (Level 2)

The city already has very good infrastructure and adequate resources for support.
For the next stage of development, the city needs to

(8) demonstrate action in linking healthy settings to promote better health;
(9) demonstrate how policies are being translated into practice and how the city makes use of its city health profile;
(10) demonstrate how local leaders and stakeholders are placed in the infrastructure of a Healthy City to push the Healthy City movement forward;
(11) launch many new initiatives to involve the community members and have established a programme to assist ‘disadvantaged’ groups (e.g., ‘New citizen’);
(12) hold many seminars on a wide range of topics to empower the citizens to lead a healthy life, and policies and community actions have been laid down to sustain the effect;
(13) develop a comprehensive set of indicators to measure the health of the population;
(14) have made progress to support a healthy environment; and
(15) hold many local and international forums to promote the concept of a Healthy City.

The city is dynamic and full of energy in new initiatives to create a supportive environment for better health.

AFHC Award for a Healthy City with Strong Action (Level 3)

(16) For advanced development of a Healthy City, the Healthy City becomes an integral part of city planning and development.
(17) The city should have resources allocated for research and development, including networking with cities nationally and internationally, taking the city into the international arena.
(18) Local politicians and leaders are well aware of the importance of a Healthy City to the livelihood of the local residents.
(19) There will be active participation from professional groups and both the academic and private sectors.
(20) The city has measures preserving natural capital and sustainable ecological system. The city’s investment in environment improvement and ecological protection is substantial (e.g., moving industrial enterprise out with tertiary industries moving in, stopping, or refusing projects with some kinds of pollution by the environment

protection departments, gathering industries in the parks and stopping the ratification of the scattered industry).<br>(21) The city has initiated the construction of the rural concentrated living quarters, and the concept of cycle economy and the economic chains of the recycling resources have been rapidly connected.

Supplementary File S3 Background of the three cities

**Gangdon-gu**<br>Gangdong District (Gangdong-gu) is one of the 25 *gu* (district) which make up the city of Seoul, South Korea. Gangdong is literally "east of the (Han) River". It is located on the east side of the city. It has population of 465,958 and area of 24.587 km^2 area with population density of 19,000/km^2.
**Owariasahi City**<br>It is located in Aichi Prefecture, Japan. As of 1 October 2019, the city had an estimated population of 81,954 in 35,583 households, and a population density of 3,897 persons per km^2. The total area of the city is 21.03 square kilometres (8.12 sq mi).
**Kwai Tsing**<br>Kwai Tsing is is one of the 18 districts of Hong Kong18 districts of Hong Kong It consists of two parts – Kwai Chung and Tsing Yi. Kwai Tsing is part of the New Territories. It had a population of 520,572 in 2016. The district has the third least educated residents and their income is below average.
